# Riverine phosphorus gain and loss across the conterminous United States

**DOI:** 10.5194/essd-18-3355-2026

**Published:** 2026-05-19

**Authors:** Yiming Wang, Xuesong Zhang, Kaiguang Zhao, Robert D. Sabo, Yuxin Miao, Christopher M. Clark

**Affiliations:** 1 Ohio Agricultural Research and Development Center, School of Environment and Natural Resources, The Ohio State University, Wooster, OH 44691, USA; 2 USDA-ARS Hydrology and Remote Sensing Laboratory, Beltsville, MD 20705-2350, USA; 3 United States Environmental Protection Agency, Office of Research and Development, Center for Public Health and Environmental Assessment, Health and Environmental Effects Assessment Division, Washington, DC 20460, USA; 4 Precision Agriculture Center, Department of Soil, Water and Climate, University of Minnesota, St. Paul, MN 55108, USA

## Abstract

Excess riverine phosphorus represents a preeminent catalyst for water quality degradation. Spatial mapping and characterization of the net gain and loss of riverine phosphorus help discern the critical source areas. Here, we developed a dataset encompassing phosphate (${\text{PO}}_{4}^{3-}$) and total phosphorus (TP) gain and loss across catchments in the conterminous United States (CONUS). We compiled 51 394 ${\text{PO}}_{4}^{3-}$ and 285 675 TP concentration measurements and estimated ${\text{PO}}_{4}^{3-}$ and TP loads at 963 and 2317 stations, respectively. Next, we leveraged the upstream-downstream topology information from the National Hydrography Dataset Plus (NHDPlus) catchment map at the Hydrologic Unit Catalogue-12 (HUC12) level to derive the net gain and loss of riverine phosphorus across catchments in the CONUS. Such maps can be used to estimate potential contributions of point and non-point sources to riverine phosphorus pollution at refined spatial scales, identify different major factors controlling local riverine P gain and loss compared to P loads, and evaluate watershed model’s fidelity for representing riverine P cycling. The resultant dataset is provided in Excel (.xlsx) format, accessible at Figshare (https://doi.org/10.6084/m9.figshare.28509317, Wang et al., 2025b). Leveraging the HUC12 information for spatialization, the new datasets aim to address the existing gap in regional characterization of riverine phosphorus and support effective management practices across the CONUS.

## 1 Introduction

Eutrophication in inland waters and estuaries is a widespread water quality challenge across the globe, with significant economic cost (e.g., USD 1 billion in Europe and USD 2.2 billion annually in the United States (U.S.)) (Wurtsbaugh et al., 2019). Excess phosphorus (P) is a primary contributor to eutrophication in streams and rivers, especially in intensive agricultural regions (Brownlie et al., 2022; Royer et al., 2006). Riverine export of P is also a major contributor to oxygen-depleted dead zones in coastal waters, causing damage to underwater life (Diaz and Rosenberg, 2008). There is an urgent need for global actions to reduce P pollution for the environment and human health (UNEP, 2025).

Nonpoint or diffuse sources, particularly nutrients applied to agroecosystems, are often recognized as the primary source of water pollution (Carpenter et al., 1998). P surplus in agricultural soils due to unused fertilization and manure application can be transported to water bodies through surface runoff and groundwater pathways, and cause persistent water pollution (Stackpoole et al., 2019). The diffuse nature of nonpoint source pollution poses challenges for directly identifying and regulating critical source areas. Existing riverine P pollution databases mainly focus on certain agricultural areas (Ringeval et al., 2024). Given the considerable spatial variation in P inputs to rivers (Arheimer and Lidén, 2000; Stackpoole et al., 2019; Zhang et al., 2017), there is a lack of large-scale riverine P datasets at sufficient spatial scales across the conterminous United States (CONUS) to quantify and analyze riverine P gain and loss. Such datasets, in conjunction with other observed and modelled P data (e.g., point source discharge) help identify regions with high non-point source P inputs, thereby supporting more effective targeting of measures for P pollution control. In addition, the datasets can also be used to assess fidelity of distributed watershed models and understand key factors influencing local riverine P cycling.

In this study, we aim to develop new datasets for spatial characterization of riverine P gain and loss across the CONUS, to help identify critical source areas and improve prioritization and implementation of nutrient management activities. The subsequent sections of this paper include the method used to generate spatial riverine P gain and loss (Sect. 2), results detailing the P dataset (Sect. 3), discussion of influencing factors and potential uncertainties (Sect. 4), codes and data availability (Sect. 5), and conclusions (Sect. 6).

## 2 Materials and Methods

### 2.1 Overview

**Figure 1.**
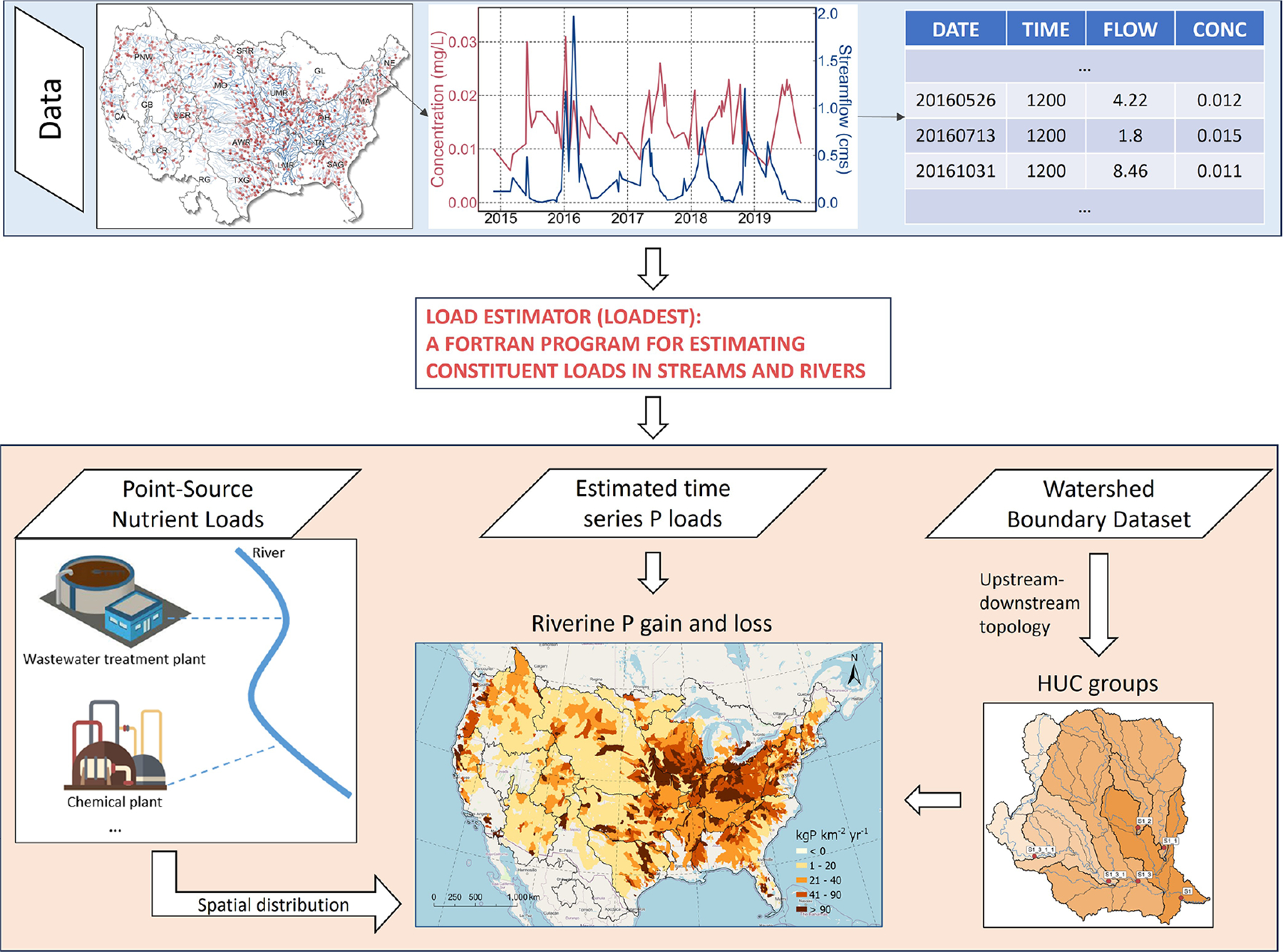
Overview of the generation of TP and ${\text{PO}}_{4}^{3-}$ gain and loss data across the CONUS. A more detailed description and higher resolution figure about HUC group generation can be found in Sect. S2 and Fig. S1.

To estimate riverine P gain and loss data across the CONUS, we compiled streamflow and P concentration data (i.e., unfiltered phosphate (${\text{PO}}_{4}^{3-}$)) from 963 monitoring stations and total phosphorus (TP) from 2317 stations, and calculated P loads (kgP yr^−1^) at these stations using the Load Estimator (LOADEST) program (Runkel et al., 2004) (Fig. 1). Next, we estimated P gain and loss across the catchments measured by one downstream station and its immediate upstream stations using the upstream-downstream connectivity information contained in the National Hydrography Dataset Plus (NHDPlus) catchments (https://nhdplus.com/NHDPlus/NHDPlusV2_data.php, last access: 7 May 2026), resulting in 547 and 1225 unique Hydrologic Unit Catalogue (HUC) groups for ${\text{PO}}_{4}^{3-}$ and TP, respectively. Each HUC group has a unique pair of downstream and upstream stations that allows us to calculate the gain and loss of riverine P (as illustrated in Fig. S1 in the Supplement). Note that headwater HUC groups only have one downstream station without upstream stations draining to them. Due to differences in data availability, the riverine ${\text{PO}}_{4}^{3-}$ and TP gain and loss data cover 52 020 and 65 735 Hydrologic Unit Catalogue-12 (HUC12) catchments, respectively. Then we estimated potential contribution to P pollution from nonpoint sources by subtracting upstream P inputs and point source P inputs from the riverine P gain and loss in each catchment. Finally, we used land cover and climate data to evaluate major controls of riverine P gain and loss.

### 2.2 Study area

The CONUS (i.e., the lower 48 states of the U.S.) is located in North America from 46°20′ to 98°34′ W longitude and from 39°49′ to 41°43′ N latitude, covering an area of 8 080 464.3 km^2^ with a north-to-south distance of approximately 2660 km. The terrain features higher elevations in the west and flatter areas in the east. Based on the Watershed Boundary Dataset (WBD; https://water.usgs.gov/GIS/huc.html, last access: 7 May 2026), the CONUS includes 18 major watersheds, encompassing several large rivers such as the Mississippi and Colorado Rivers.

### 2.3 Data compilation

We compiled 51 394 ${\text{PO}}_{4}^{3-}$ (USGS parameter code 00650) concentration data from 963 stations (spanning from 1952 to 2022) and 285 675 TP (USGS parameter code 00665) observations from 2317 stations (spanning from 1958 to 2023) across the CONUS from the Water Quality Portal (Read et al., 2017). To ensure consistency with streamflow records, only records from the U.S. Geological Survey (USGS) National Water Information System (NWIS) data source were used in this study. For each P observation, we identified co-located monitoring stations (Wang et al., 2024b) and downloaded and processed daily streamflow data from the USGS NWIS. Only stations with both phosphorus concentration observations and corresponding daily streamflow records were retained. We calculated an average P concentration where there were multiple P concentration observations on the same day. Before calculating the P load at a station with the LOADEST model, we excluded stations with less than 12 measurements. Thus, we finally selected 547 stations for ${\text{PO}}_{4}^{3-}$ and 1225 stations for TP. For TP, the “Point-Source Nutrient Loads to Streams of the Conterminous United States” dataset provides estimated annual total point-source inputs during 2012 at the HUC12 level (Skinner and Maupin, 2019). This allowed us to aggregate the total TP input to rivers for the catchments used to calculate riverine TP gain and loss. Note that the point source dataset does not contain ${\text{PO}}_{4}^{3-}$.

Land cover and climatic controls of riverine P gain and loss were also assessed. Land cover data were derived from the National Land Cover Database (NLCD; https://doi.org/10.5066/P94UXNTS, USGS, 2024), which provided long-term average information on various land cover types: barren land, crops, forest, hay, herbs, impervious surfaces, scrub, water, herbaceous wetlands, and woody wetlands (Homer et al., 2012). Climate data were sourced from the PRISM dataset (https://www.prism.oregonstate.edu/, last access: 7 May 2026), including annual average temperature and total precipitation (Page et al., 2021). Using upstream-downstream topology information, we calculated the total area of each land cover type from the headwater to the current catchment at the HUC12 scale, representing their cumulative impact. For climate data, the local climate within each HUC12 was used. The P surplus was accessed from the National Inventory of Phosphorus (NIP), which provides major inputs and outputs of reactive P at the HUC8 scale across the CONUS (Sabo et al., 2021).

To ensure consistency across datasets with different spatial resolutions, all analyses were anchored at the HUC12 group scale. Point-source inputs, originally reported at the HUC12 level, were aggregated to HUC12 groups to match the gain-loss estimates. For datasets available at coarser scales (e.g., HUC8 for NIP inputs and HUC4 for agricultural inputs), gain and loss were upscaled using area-weighted averaging. No downscaling was applied.

### 2.4 Riverine P gain and loss across catchments in the CONUS

Riverine P gain and loss was estimated by calculating the difference between P loads at a downstream monitoring station and the sum of P loads from its neighbouring upstream stations. For multiple USGS stations located in the same HUC12 catchment, we kept only one station on the mainstem of the river that is closest to the outlet of the HUC12 catchment, by comparing the drainage area of the gaging station and the HUC12 catchment in which it is located. For headwaters, since there are no upstream gages, the P load was used as the net riverine gain. The upstream-downstream topology relationship between the monitoring stations was derived from the HUC12 catchments from the watershed boundary dataset (WBD; https://water.usgs.gov/GIS/huc.html, last access: 7 May 2026). Such a method has been outlined and tested by Qiu et al. (2023) (Sect. S2). As explained above, we identified 547 and 1225 unique HUC groups for ${\text{PO}}_{4}^{3-}$ and TP, respectively. Note that each HUC group includes multiple HUC12 polygons and these HUC12 catchments share the same gain and loss data.

For each HUC group, the balance of riverine P can be expressed as follows:

(1) Ploadatdownstreamoutlet=(Ploadsfromupstreaminputs)+(Pfrompointsources)-(RiverineremovalofPfrompointsources)+(Pfromnon-pointsources)-(RiverineremovalofPfromnon-pointsources)

Because in-stream removal terms cannot be directly constrained and may vary across systems (e.g., about 12% globally) (Maavara et al., 2015), we assume the values of these two terms to be negligible for the purpose of deriving a simplified, first-order estimate for nonpoint source inputs. Thus, riverine removal is not explicitly estimated in this study, and the calculated P gain and loss represent the net difference between downstream and upstream loads. Conceptually, this net difference reflects the combined effects of watershed P inputs (from both point and nonpoint sources) and in-stream processes (e.g., retention, transformation, and remobilization) occurring along the flow path, rather than a direct measure of any single process such as in-stream removal. Under this assumption, Eq. (1) reduces to:

(2) (Pfromnon-pointsources)=Pgainandloss-(Pfrompointsources)

where P gain and loss = (P load at downstream outlet) − (P loads from upstream inputs).

Accordingly, negative values of P gain and loss indicate net decreases in load along the flow path, which may reflect a combination of retention, transformation, or other processes, rather than being interpreted solely as riverine removal. This formulation neglects in-stream P removal, and therefore (P from nonpoint sources) = P gain and loss − (P from point sources) is a lower-end estimate of the nonpoint source contribution to riverine P for each HUC group. Since only TP from point sources is available, we derived nonpoint-source TP loads but not for ${\text{PO}}_{4}^{3-}$.

### 2.5 Evaluation of estimated riverine load

We evaluated the consistency of the ${\text{PO}}_{4}^{3-}$ and TP loads against another independent dataset derived with the Weighted Regressions on Time, Discharge, and Season (WRTDS) model (Hirsch et al., 2010; Zhang and Hirsch, 2019). First, we compared multi-year average TP loads from 151 monitoring stations. We also evaluated the estimated unfiltered ${\text{PO}}_{4}^{3-}$ loads, which assess the mass of reactive P susceptible to being released in the water column under various redox conditions. Furthermore, the reliability of the upstream-downstream connectivity information is important for deriving the drainage area of HUC groups that are controlled by pairs of upstream and downstream stations. Here we used a quality-checked and corrected NHDPlus HUC12 catchment map (Wang et al., 2024a) that has been verified for reliably deriving the drainage area of each USGS station as compared to the USGS GAGES-II reported values (Falcone and Survey, 2011). These efforts helped ensure the quality of the riverine P gain and loss data developed in this study. In addition, we evaluated long-term trends in riverine P loads using the Sen’s slope estimator in combination with the Mann–Kendall test (Hamed and Rao, 1998). To ensure robust trend detection, only monitoring stations with more than 30 years of load estimates were included. This resulted in 405 TP stations and 53 ${\text{PO}}_{4}^{3-}$ stations used for trend analysis. The Sen’s slope and corresponding p-values are provided in the dataset.

### 2.6 Analysis of environmental controls

Recent studies reveal that shifts in land use, agricultural practices, and climatic conditions have introduced a pervasive increase in soluble P concentrations across many different watersheds (Houser and Richardson, 2010; Singh et al., 2023). To assess the spatial factors influencing riverine P gain and loss, we employed random forest modelling to evaluate the relative importance of multiple environmental variables (Breiman, 2001). These factors were categorized into three groups: climatic factors, land cover types, and additional influences such as cumulative agricultural inputs and upstream loads. Given that the NIP dataset is only available at the HUC8 scale and some HUC groups are larger than the HUC8 catchment areas, we calculated the cumulative agricultural inputs at the HUC4 scale. In more detail, we used the ranger package, optimizing the model structure with the caret package in R. Key tuning parameters included the number of variables to use in each split (mtry), the number of trees (n_trees) and the minimum size of data points before splitting a tree (min_n). The tuning process was performed by doing a grid search for mtry (2–6) and min_n (10–20), then a second search was performed to find the optimal n_trees parameter (500–3000). To minimize random effects, the model was run 10 times, and we calculated the average importance value and harmonic mean p-value (Wilson, 2019).

## 3 Results

### 3.1 Riverine phosphorus data

We created two datasets, “Riverine ${\text{PO}}_{4}^{3-}$” and “Riverine TP”, that encapsulate estimated multi-year average riverine gain and loss and loads, as well as point source and nonpoint source contributions for each HUC group for ${\text{PO}}_{4}^{3-}$ and TP, respectively (Table 1). Complementing this information, the datasets encompass the location (i.e., longitude and latitude) of the outlet of the HUC group, the area of the HUC group, the count of observations used to calculate P loads, and commencement and termination years of observed data, to facilitate user-defined subsetting of the datasets. Additional information regarding the regression model is also included, such as the form of the regression model selected by LOADEST and the associated coefficient of determination ($r^{2}$) values.

Across the board, the average $r^{2}$ values for the best-fit model (Table S1) across all sites are 0.76 for ${\text{PO}}_{4}^{3-}$ loads and 0.83 for TP loads. Generally, in the load regression, the $r^{2}$ values for TP estimation outperformed those for ${\text{PO}}_{4}^{3-}$ estimation, particularly in the Mid-Atlantic region (Fig. S2). It is noteworthy, however, that certain monitoring stations exhibited low $r^{2}$ values due to the limited availability of paired P concentration with streamflow data for regression.

**Figure 2.**
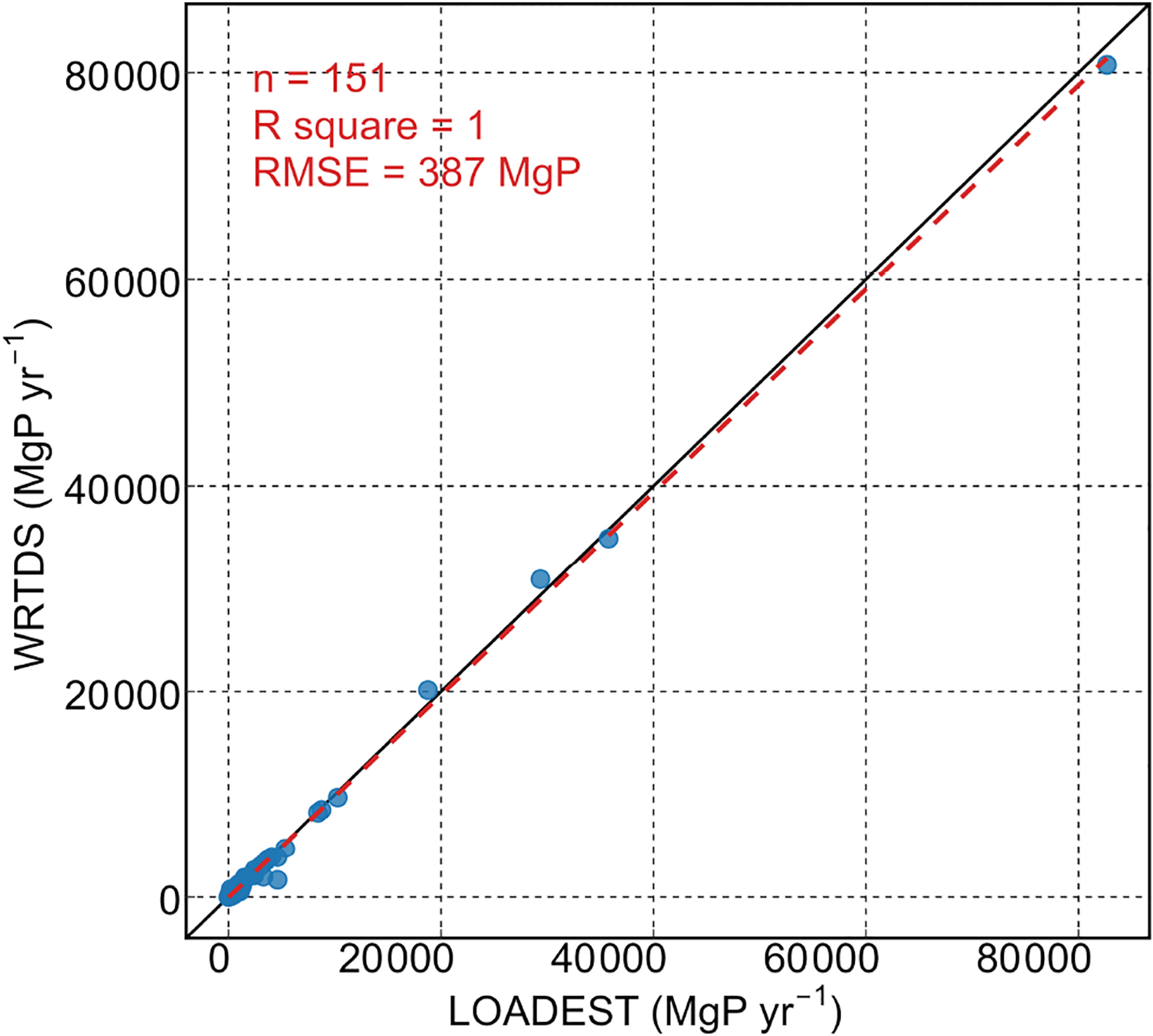
Comparison of riverine TP loads between our estimates and previous WRTDS estimated values at 151 monitoring stations. Note that the riverine TP loads vary greatly at different locations and over time, with most sites being below 10 000 MgP yr^−1^.

Our TP load estimates are highly consistent with the WRTDS model with high $r^{2}$ and low root mean square error (RMSE) (Fig. 2). The minor disparities observed between these two datasets are likely attributable to variations in temporal coverage and different regression equations. For ${\text{PO}}_{4}^{3-}$, we found that only 11 stations with WRTDS estimates matched the stations used here, and all 11 stations are located in small watersheds. Therefore, we leveraged filtered ${\text{PO}}_{4}^{3-}$ loads estimated by WRTDS to assess if the LOADEST estimated unfiltered ${\text{PO}}_{4}^{3-}$ loads. Unfiltered ${\text{PO}}_{4}^{3-}$ measures both the dissolved ${\text{PO}}_{4}^{3-}$ as well as ${\text{PO}}_{4}^{3-}$ compounds bound to suspended sediments and organic materials, and thus will have higher load compared to filtered ${\text{PO}}_{4}^{3-}$ measurements (Fig. S3). Nonetheless, the high correlation indicates our estimates of unfiltered ${\text{PO}}_{4}^{3-}$ are reasonable.

**Figure 3.**
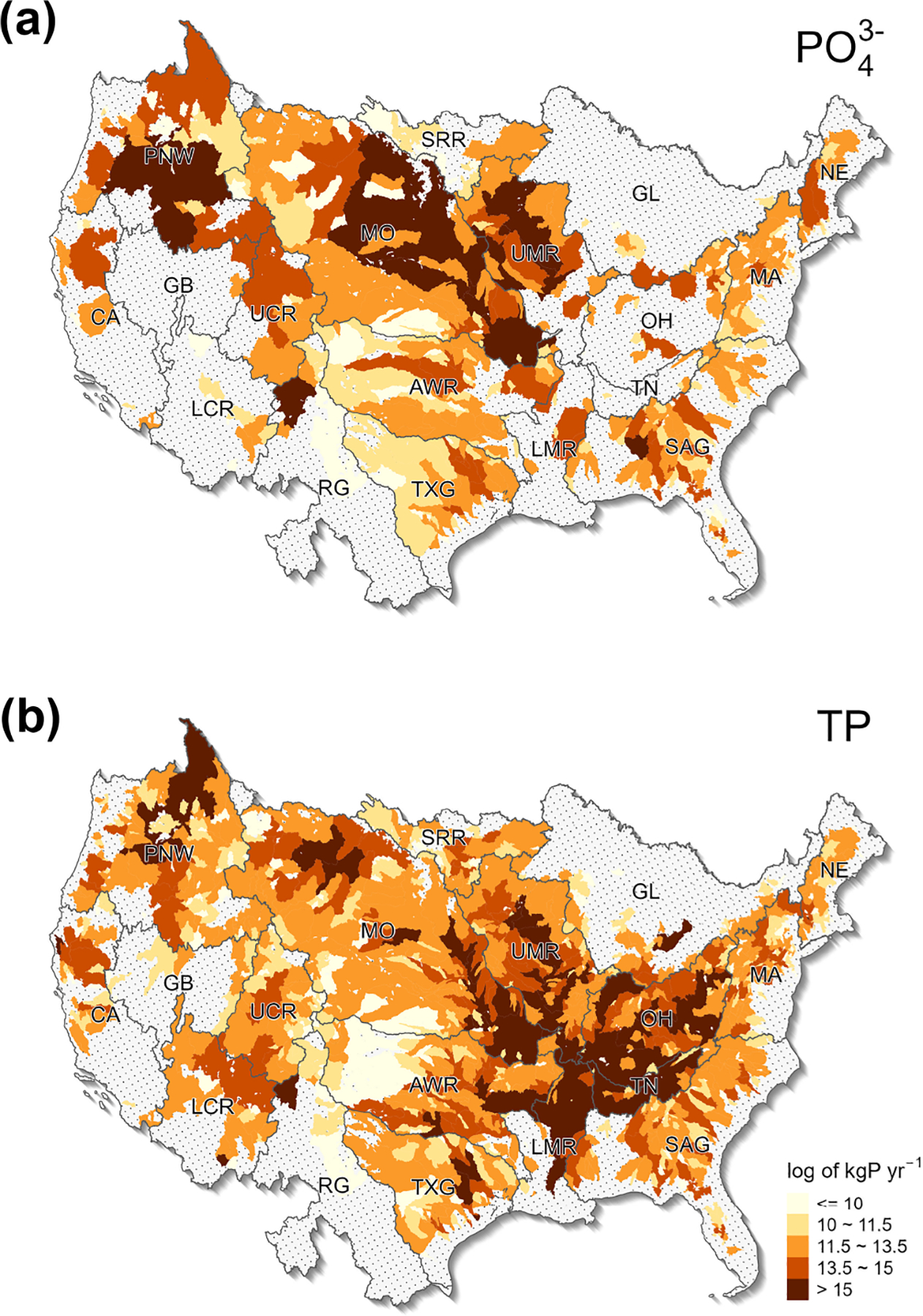
Riverine **(a)** ${\text{PO}}_{4}^{3-}$ and **(b)** TP loads from HUC groups across the CONUS. The boundary lines show the Hydrologic Unit Catalogue 2-digit (HUC2) watersheds. Grey areas indicate regions with no data. For visualization purposes, the logarithm was used here.

Spatial patterns of ${\text{PO}}_{4}^{3-}$ and TP loads from each HUC group across the CONUS are shown in Fig. 3. Additionally, the location of the station at the outlet of each HUC group and the loads of streams in which it is located are shown in Fig. S4. The datasets encompass 547 stations/HUC groups for ${\text{PO}}_{4}^{3-}$ and 1225 stations/HUC groups for TP, covering 4 894 464 and 6 118 360 km^2 ${\text{PO}}_{4}^{3-}$^ and TP, respectively. At the HUC2 scale, the derived gain and loss estimates cover 21% to 99.9% of basin area for TP and 7% to 96.5% for ${\text{PO}}_{4}^{3-}$, with 72% and 44% of HUC2 basins exceeding 50% coverage, respectively (Fig. S5 and Table S2). The difference in spatial coverage is mainly due to the abundance of TP compared to ${\text{PO}}_{4}^{3-}$. Stations with high P loads are predominantly situated in the Midwest or proximate to megacities, with a general pattern of higher P loads observed in the eastern U.S. ${\text{PO}}_{4}^{3-}$ loads range from 111 to 31 671 885 kgP yr^−1^ and TP loads range from 235 to 336 223 136 kgP yr^−1^. Median loads are 76 202 and 108 305 kgP yr^−1^ and average loads are 436 311 and 1 012 363 kgP yr^−1^, for ${\text{PO}}_{4}^{3-}$ and TP, respectively.

We further examined temporal trends in riverine P loads at stations with long-term records (>30 years). A substantial fraction of stations exhibited statistically significant trends, with 46% of TP stations and 64% of ${\text{PO}}_{4}^{3-}$ stations showing significant changes. Decreasing trends were more prevalent, accounting for 72% and 58% of TP and ${\text{PO}}_{4}^{3-}$ stations, respectively. Stations with increasing TP trends were primarily located in the Mississippi River Basin (Fig. S6), suggesting regional differences in nutrient dynamics.

**Figure 4.**
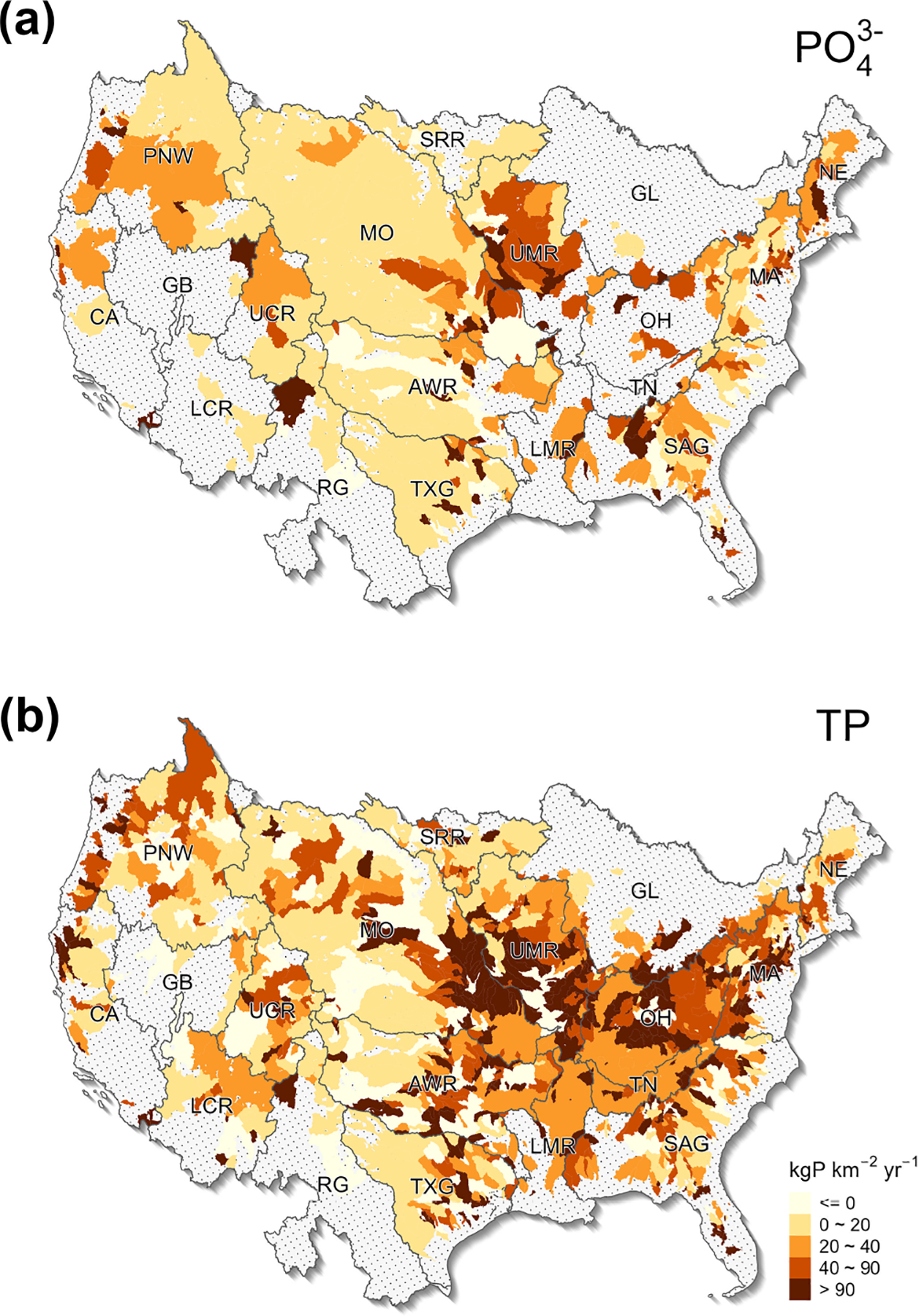
The spatial distribution of the gain and loss in riverine **(a)** ${\text{PO}}_{4}^{3-}$ and **(b)** TP over the CONUS. The boundary lines show the Hydrologic Unit Catalogue 2-digit (HUC2) watersheds. Grey areas indicate regions with no data.

The spatial distribution of riverine P gain and loss is shown in Fig. 4. Both ${\text{PO}}_{4}^{3-}$ and TP gain and loss exhibit similar spatial patterns over the CONUS, with most areas exhibiting riverine P gains. The area-weighted average ${\text{PO}}_{4}^{3-}$ gain stands at 25.39 kgP km^−2^ yr^−1^, and the TP gain is 33.68 kgP km^−2^ yr^−1^. Median ${\text{PO}}_{4}^{3-}$ and TP gains are lower than averages, standing at 16.75 and 33.57 kgP km^−2^ yr^−1^, respectively. At the HUC group scale, the highest area-weighted ${\text{PO}}_{4}^{3-}$ gain was identified in the Upper Mississippi Region (UMR), amounting to about 113.96 kgP km^−2^ yr^−1^. The highest TP gain reached 186.55 kgP km^−2^ yr^−1^ in the Tennessee Region (TN). Notably, widespread regions in the Midwest exhibit heightened P gains (Fig. S7), particularly in terms of ${\text{PO}}_{4}^{3-}$, suggesting a discernible impact of human activities (e.g., agricultural fertilization). At the HUC2 level, the lowest area-weighted ${\text{PO}}_{4}^{3-}$ gain (1.72 kgP km^−2^ yr^−1^) was found in the Rio Grande Region (RG), and the lowest TP gain (0.62 kgP km^−2^ yr^−1^) was found in the Upper Colorado Region (UCR). Refined examination at the HUC group level showed that, over the CONUS, 392 778 and 1 468 973km^2^ areas exhibited riverine ${\text{PO}}_{4}^{3-}$ and TP losses, respectively.

### 3.2 Point and nonpoint source contributions

**Figure 5.**
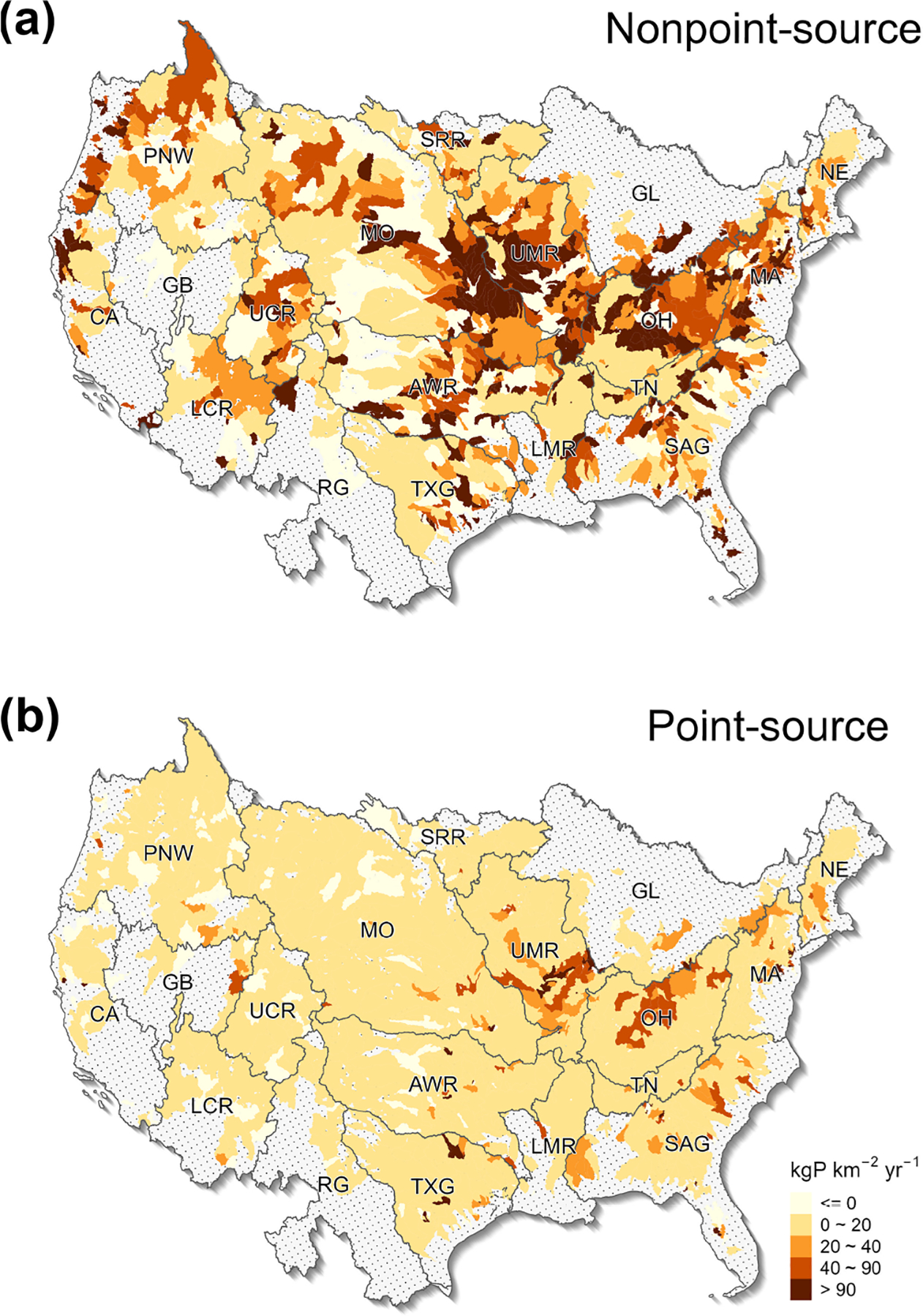
The spatial distribution of **(a)** nonpoint source and **(b)** point source contributions to riverine P pollution over the CONUS. The boundary lines show the Hydrologic Unit Catalogue 2-digit (HUC2) watersheds. Grey areas indicate regions with no data.

We also mapped the spatial point source and nonpoint source inputs of TP, as shown in Fig. 5. The nonpoint source contributions are estimated based on Eq. (2), which provides a lower-end estimate given that the riverine removal of point and nonpoint source P as shown in Eq. (2) was not considered. Point and nonpoint source contributions to riverine TP pollution exhibited large differences in both the magnitude and spatial distribution. Over the CONUS, the area-averaged point source input of TP is 5.44 kgP km^−2^ yr^−1^. By subtracting point source inputs from the calculated TP gain and loss, we obtained an area-averaged nonpoint source TP contribution of 28.24 kgP km^−2^ yr^−1^. Regions characterized by high TP gain with minimal point source pollution were observed in the Midwest. Notably, in most of the agriculturally intensive Missouri and Tennessee-Ohio river basins, total nonpoint source discharge significantly surpassed point source contributions (Fig. S8). Upon the exclusion of point source contributions (Fig. 5b), there is a substantial change, with the areas with riverine TP losses expanding to 1 603 258 km^2^, most of them in the Missouri and Arkansas-White-Red River Basins. In general, most watersheds with negative nonpoint sources are concentrated in the western U.S. This does not mean that the nonpoint source P inputs are negative, but indicates that riverine processes likely removed a large fraction of point and nonpoint source P.

### 3.3 Factors influencing TP

**Figure 6.**
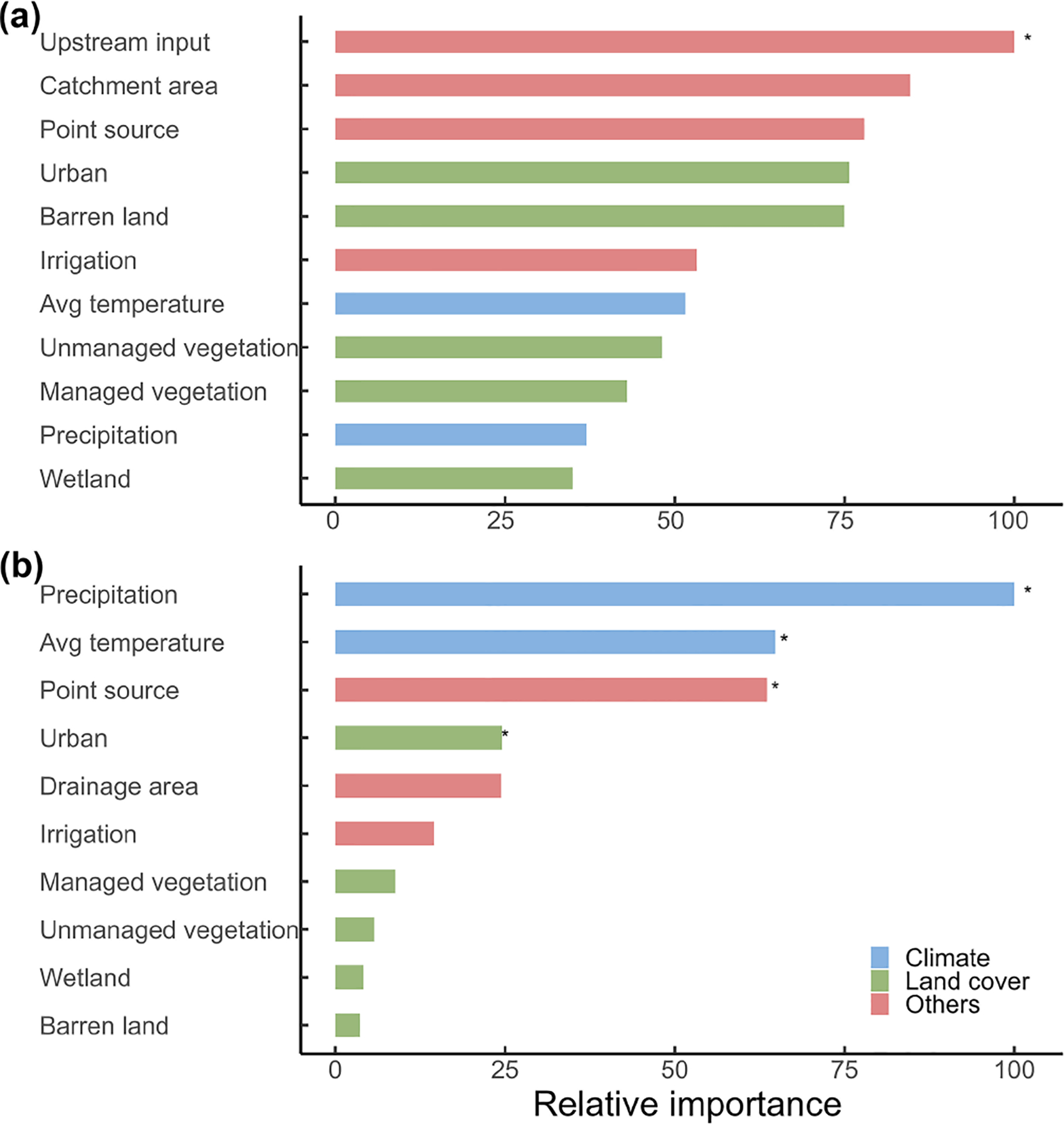
The importance of factors influencing **(a)** TP gain and loss and **(b)** TP loads. Asterisks indicate significance at a level of 0.05.

We employed a random forest model to assess the influence of climate, land use, human activities, and catchment characteristics on riverine TP gain and loss and TP loads (Fig. 6). Note that the calculated P loads represent the outcome of the entire upstream catchment processes, while the riverine P gain and loss data represent both upstream catchment processes (e.g., P inputs from upstream) and local catchment properties (e.g., climate and land use in a HUC group). Such differences lead to the use of different sets of influencing factors (Fig. 6). The land use, climate and point source factors were calculated for the entire upstream area draining to a monitoring station for TP load analysis. In contrast, those factors were averaged over a HUC group for riverine gain and loss analysis. Additionally, for the analysis of riverine P gain and loss data, we included upstream P inputs. Analysis results indicate that upstream input is the sole statistically significant factor affecting TP gain and loss, with climate and land cover showing no notable impact. Conversely, TP loads are predominantly influenced by climatic factors, alongside significant contributions from point source discharges and urban land use.

## 4 Discussion

### 4.1 Important contributions from nonpoint sources to riverine P pollution

The estimated area-averaged nonpoint source TP contribution (28.24 kgP km^−2^ yr^−1^) represents a reduction of 16.2% from the calculated TP gain and loss that includes contributions from both point and nonpoint sources (33.68 kgP km^−2^ yr^−1^). Given that the TP inputs from point and nonpoint sources are often subject to riverine removal (Withers and Jarvie, 2008), the estimated nonpoint source TP based on Eq. (1) should be augmented by the amount of total TP inputs (including both nonpoint and point source) removed through riverine processes. Therefore, the calculated nonpoint source inputs of TP represent an underestimate of the contributions from nonpoint sources. If we assume a 12% removal rate for TP inputs (Maavara et al., 2015), then the nonpoint source inputs of TP would increase from 28.24 to 32.28 kgP km^−2^ yr^−1^. Collectively, the results show that the nonpoint sources likely contribute more than 84% of riverine TP pollution.

### 4.2 Implications for analysing environmental controls of riverine P

Climatic factors were key drivers of TP loads at the outlet of a watershed, which in general aligns with findings from previous studies (Sabo et al., 2023) and underscores the role of climate in nutrient transport dynamics. Our environmental control analysis using the gain and loss data showed that upstream inputs are leading control of local riverine gain and loss (Fig. 6a), in addition to local inputs of P from point and nonpoint sources and local riverine processes, such as in-stream retention through mechanisms such as P absorption by periphyton via photosynthesis and hydrological processes like reduced streamflow and sedimentation (Dodds, 2003; Withers and Jarvie, 2008). Notably, although accumulated agricultural P inputs (i.e., livestock waste and agricultural fertilizer) positively influenced TP gain and loss (Fig. S9), they were not included in this analysis due to mismatch of spatial scales. In general, using TP loads and riverine P gain and loss can lead to pronounced differences in the analysis of importance of environmental controls. Although climate conditions (i.e., precipitation and temperature) are the major controls of TP loads, which represent the integration of the entire watershed conditions, while riverine P gain and loss indicate that the amount of upstream P inputs entering a local catchment is an important factor influencing the riverine processing of P.

Both the differences and the analysis using TP loads and riverine gain and loss data revealed the importance of urban land and agricultural management. For example, both analyses show that irrigation can influence riverine TP, indicating that improving irrigation efficiency and technology holds potential to reduce TP inputs from cropland fertilization (Xia et al., 2020). Though we didn’t assess factors influencing ${\text{PO}}_{4}^{3-}$ due to the lack of point source ${\text{PO}}_{4}^{3-}$ data, TP hotspots are expected to occur further downstream than ${\text{PO}}_{4}^{3-}$ hotspots (Fig. 3). This is probably because rivers typically can retain (e.g., periphyton assimilation, adsorption onto suspended or bed sediment) a considerable proportion of incoming soluble-reactive P (e.g., ${\text{PO}}_{4}^{3-}$) within the upper network, whereas particulate P continue to transport to downstream (Jarvie et al., 2012; Robertson and Saad, 2019; Royer et al., 2006). Overall, the intricate interplay between climate and land use factors underscores the complex nature of P dynamics in riverine systems. These newly developed riverine gain and loss datasets help improve understanding of local controls of riverine P dynamics and identify hotspots of changes in riverine P.

### 4.3 Limitations and contribution

While the newly developed datasets leverage upstream-downstream topology information at the HUC12 level to help increase the spatial resolution of riverine P gain and loss data, it is essential to acknowledge limitations relevant to understanding and quantifying P cycles and identifying sources of P inputs. First, the accuracy of load estimation via LOADEST is contingent upon the availability of paired P concentration data from monitoring stations. Stations with limited observations may introduce higher uncertainties in load estimations. The robustness of our datasets is partially reflected in the number of observations available. The average and median numbers of ${\text{PO}}_{4}^{3-}$ observations per site is 54 and 29, respectively; for TP, the average and median numbers of observations per site is 134 and 90, respectively. In addition, the average center year of TP observations is 1992, with a median of 1991, while for ${\text{PO}}_{4}^{3-}$, the overall observation time is relatively early, with an average of 1981 and a median of 1980. The use of AIC to choose the most parsimonious regression model (average $r^{2}$ of 0.76 and 0.83 for ${\text{PO}}_{4}^{3-}$ and TP, respectively) helped reduce uncertainties in load estimates.

Additionally, we calculated the P loads by averaging over different time periods with available data for each monitoring station. The mismatch between observational periods of upstream and downstream stations could introduce uncertainties, given that the available data cover various time periods for different stations. For example, streamflow discharge, which is important for calculating nutrient loads, can vary from year to year. Here, we assumed that multi-year average estimates of P loads are representative of the long-term pattern at a monitoring station. This may not hold for some upstream and downstream stations covering time periods that do not overlap with each other. Therefore, we provided the number and period of observations and model performance information in the datasets, which can help users to refine the calculation of riverine P gain and loss by further screening the P loads data at the monitoring stations. Note that available stations with observed P concentration and streamflow data are relatively sparse in the western vs. eastern U.S., particularly for ${\text{PO}}_{4}^{3-}$. This led to large gaps in the spatial coverage of the datasets (Fig. 4). Increasing the number of stations with P observations holds the potential to enhance the accuracy of riverine P estimates in the future.

It is also worth noting that the calculated contribution of TP from nonpoint sources represents a conservative estimate, given the unknown rate of TP removal from point and nonpoint sources. Although previous studies showed that TP removal rates were generally small, they could vary substantially across regions, as evidenced by the areas with riverine P loss (Fig. 4). It is reasonable to assume that the estimated TP contribution from nonpoint sources is greater than 84% over the CONUS; however, the local TP contribution from nonpoint sources could be much lower, particularly in regions with high point source inputs. Also, the removal rates of P from point and nonpoint sources are likely different due to differences in the quality of P inputs (e.g., biodegradability and adsorption and desorption to sediments) and flow pathways (Wang et al., 2025a). Therefore, caution should be taken when interpreting the local contribution to P pollution from nonpoint sources.

Despite these challenges, our datasets make a unique contribution to the quantification and analysis of riverine P load, gain and loss, and sources across the CONUS. They can support the evaluation and diagnosis of large-scale dynamic watershed models, the examination of environmental controls on riverine P loads, and the estimation of contributions to P gain and loss. For instance, models such as SPARROW could incorporate the spatial patterns of riverine P gain and loss to better constrain nutrient sources and in-stream processing across river networks. The insights derived from our datasets contribute to a more comprehensive understanding of P dynamics under long-term and multi-decadal conditions, providing a foundation for improved water quality management on local, regional, and national scales.

## 5 Code and data availability

All codes for validating and visualizing ${\text{PO}}_{4}^{3-}$ and TP gain and loss from the load estimations were run in R version 4.3.1 and are archived with the datasets presented in the paper at https://doi.org/10.6084/m9.figshare.28509317 (Wang et al., 2025b).

## 6 Conclusions

In this study, we estimated riverine loads of ${\text{PO}}_{4}^{3-}$ and TP and derived their gain and loss across the CONUS, leveraging the upstream-downstream hydrological connectivity information contained in the NHDPlus catchment map. On average, rivers across the CONUS gain TP at a rate of 33.68 kgP km^−2^ yr^−1^, with notable hotspots in the Midwest. Due to the limitations of data availability, the precision of estimated P gain and loss data could be influenced by the number and periods of observations available at upstream and downstream stations. We provided additional information regarding the number of observations available, temporal coverage of data, the regression model used, and the model’s statistical performance, so that users can further subset the datasets to meet certain specific criteria.

The riverine P gain and loss datasets allow the estimation of riverine P removal or accrual at a refined spatial resolution to better reflect the impacts of local controls. In contrast, riverine P loads at monitoring stations embody the integrated processes from the entire area upstream of a specific station. Also, by combining point source inputs with the riverine P gain and loss datasets, we derived conservative estimates of the long-term average contribution of nonpoint sources to riverine TP (28.24 kgP km^−2^ yr^−1^). The control factor analysis with a random forest model demonstrated that upstream inputs had the greatest influence on the local riverine P gain and loss, while climatic factors dominated riverine P loads at monitoring stations. This suggests that nutrient management practices that prioritize enhancing irrigation efficiency and integrating strategies such as targeted fertilizer application and wetland restoration may more effectively capture and reduce phosphorus mobilization from agricultural lands. The newly developed riverine P datasets in this study extend utility to diverse applications, encompassing, but not limited to, the evaluation of watershed models, identification of critical source areas, and optimization of agricultural management strategies. Future studies may concentrate on filling gaps in the spatial and temporal coverage of the datasets (particularly for ${\text{PO}}_{4}^{3-}$).

## Supplementary Material

Supplement1

**Supplement.** The supplement related to this article is available online at https://doi.org/10.5194/essd-18-3355-2026-supplement.

## Figures and Tables

**Table 1.** Data records in the “Riverine ${\text{PO}}_{4}^{3-}$” and “Riverine TP” datasets.

Field name	Description
Station ID	U.S. Geological Survey designated ID; Note that this ID is also used to denote a unique HUC group
Lat	Latitude of the monitoring station at the outlet of a HUC group
Long	Longitude of the monitoring station at the outlet of a HUC group
Area	Area of the HUC group
Load	The amount of ${\text{PO}}_{4}^{3-}$ or TP loads at the outlet station of a HUC group (kgP yr^−1^)
P gain and loss	The difference between P loads at the outlet of a HUC group and all its immediate upstream stations (kgP km^−2^ yr^−1^)
NonPts TP contribution*	Nonpoint-source contribution to riverine phosphorus from a HUC group (kgP km^−2^ yr^−1^)
Pts TP load	Point-source TP loads (kg yr^−1^) from a HUC group
obsNum	The number of phosphorus concentration data with paired streamflow data
startYr	The starting year of the observed data
endYr	The ending year of the observed data
modelID	The ID of regression model used for load estimation
r2	R-Squared (%) for the selected LOADEST regression model used to estimate the P load
Sen’s slope	Sen’s slope of riverine P loads, representing the long-term rate of change in loads at monitoring stations (kg yr^−2^).
p-value	The p-value of long-term trends
Areal-normalized slope	Areal-normalized Sen’s slope of riverine P loads, representing the long-term rate of change in areal P export (kg km^−2^ yr^−2^).

Note:

* only for TP as only point source TP inputs are available.
