# Supplementary material for: Riverine phosphorus gain and loss across the conterminous United States
Source: Earth Syst Sci Data. Author manuscript; Available in PMC 2026 Jun 16. (PMC13267132; doi:10.5194/essd-18-3355-2026)
Supplement: Supplement1 [file NIHMS2182165-supplement-Supplement1.pdf]

Supplement of Earth Syst. Sci. Data, 18, 3355–3365, 2026
https://doi.org/10.5194/essd-18-3355-2026-supplement
© Author(s) 2026. CC BY 4.0 License.

Open Access Earth System Science Data

Supplement of

Riverine phosphorus gain and loss across the conterminous United States

Yiming Wang et al.

Correspondence to: Yiming Wang (wang.20415@osu.edu) and Xuesong Zhang (xuesong.zhang@usda.gov)

The copyright of individual parts of the supplement might differ from the article licence.

S1. Estimation of riverine phosphorus load

The LOADEST, a FORTRAN program designed for estimating constituent loads in streams and rivers through Adjusted Maximum Likelihood Estimation (AMLE), was used to calculate PO_4^{3-} and TP loads at each hydrological station (Runkel et al., 2004). The Akaike Information Criteria (AIC), which evaluates both maximum likelihood and the number of independently adjusted parameters within each model (Akaike, 1974), was utilized for model selection among nine predefined regression models included in LOADEST (Table S1). A total of 935,069 PO_4^{3-} and 1,220,744 TP observations were compiled as inputs for the LOADEST model. The time span and the number of P observations at each station are provided in the shared dataset.

Table S1. Regression models in LOADEST

ID	Regression model
1	$a_0 + a_1 \ln \ln Q$
2	$a_0 + a_1 \ln \ln Q + a_2 \ln \ln Q^2$
3	$a_0 + a_1 \ln \ln Q + a_2 dtime$
4	$a_0 + a_1 \ln \ln Q + a_2 \sin \sin (2\pi dtime) + a_3 \cos \cos (2\pi dtime)$
5	$a_0 + a_1 \ln \ln Q + a_2 \ln \ln Q^2 + a_3 dtime$
6	$a_0 + a_1 \ln \ln Q + a_2 \ln \ln Q^2 + a_3 \sin \sin (2\pi dtime) + a_4 \cos \cos (2\pi dtime)$
7	$a_0 + a_1 \ln \ln Q + a_2 \sin \sin (2\pi dtime) + a_3 \cos \cos (2\pi dtime) + a_4 dtime$
8	$a_0 + a_1 \ln \ln Q + a_2 \ln \ln Q^2 + a_3 \sin \sin (2\pi dtime) + a_4 \cos \cos (2\pi dtime) + a_5 dtime$
9	$a_0 + a_1 \ln \ln Q + a_2 \ln \ln Q^2 + a_3 \sin \sin (2\pi dtime) + a_4 \cos \cos (2\pi dtime) + a_5 dtime + a_6 dtime^2$

where $\ln \ln Q$ is the difference between $\ln \ln streamflow$ and $center\ of\ \ln (streamflow)$; $dtime$ is the difference between $decimal\ time$ and $center\ of\ decimal\ time$; a_0, a_2, a_3, a_4, a_5, and a_6 are model regression coefficients.

S2. Generation of HUC groups

To generate the spatial HUC groups for the CONUS based on the locations of HUC12 catchments and USGS stations, the specific HUC12 in which each hydrological station is located was first identified, with the most downstream station designated as S1 (the first level or the most downstream station) (Fig. S1). Subsequent tracing of upstream HUC12s was conducted until reaching one or more HUC12 catchments encompassing a hydrological station. These upstream HUC12 catchments and hydrological stations were identified as the second level (e.g., S1_1 and S1_2). The HUC12s within the larger catchment of the first-level station (S1) and the second-level stations (S1_1, S1_2, etc.) were collectively grouped as the "S1" HUC group. The riverine phosphorus in the "S1" HUC group was computed by dividing the difference between the load at the first-level station and the aggregated load from the second-level stations by the area of the "S1" HUC group. This procedure was repeated by designating each hydrological station from the second-level stations as a first-level station, until all hydrological stations were processed (i.e., assigned as a first level station).

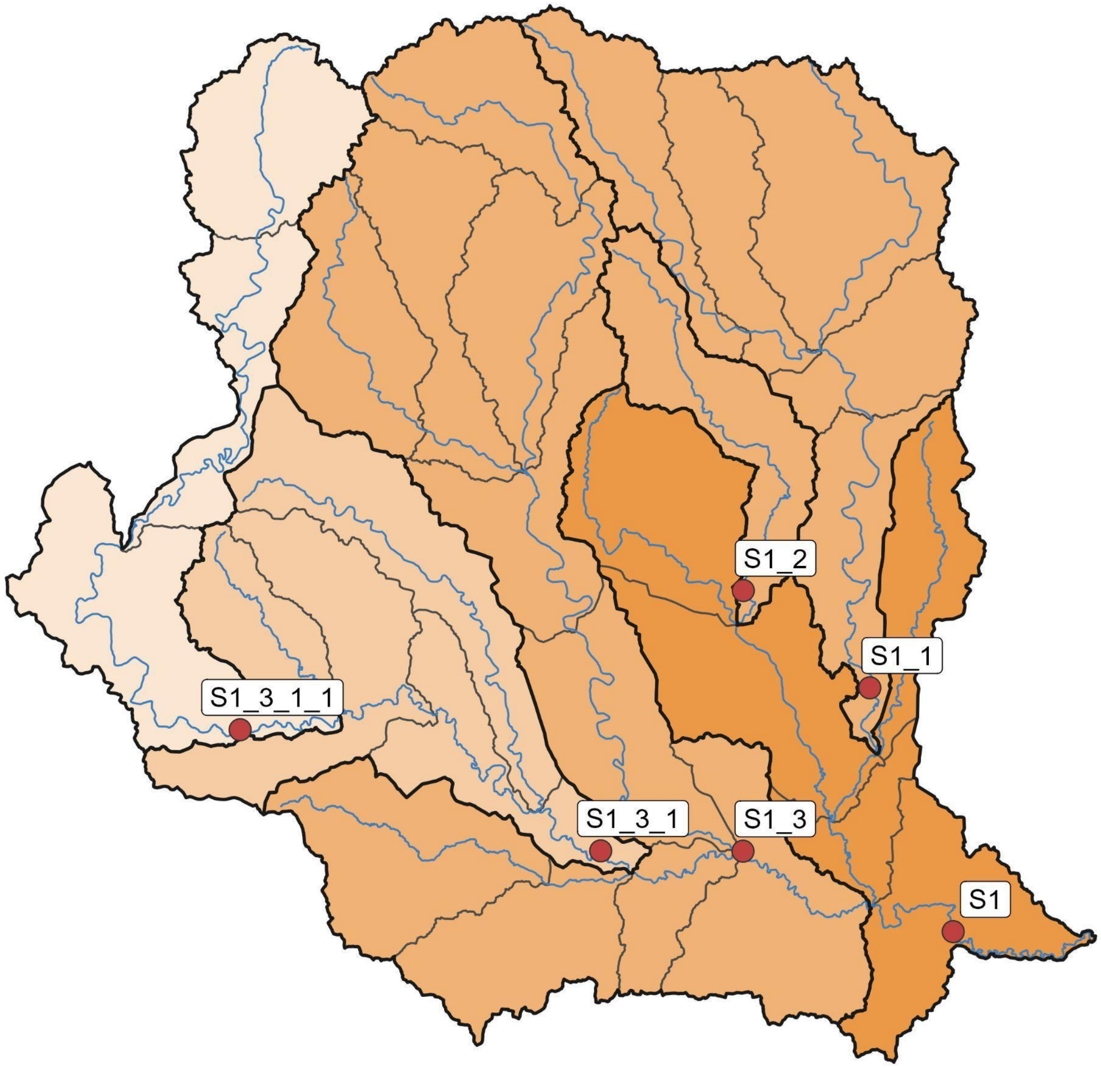

Figure S1: Illustration of HUC group generation using a watershed the San Antonio River in Texas-Gulf Region. Red dots represent 6 gaging stations; polygons with grey outlines indicate 28 HUC12 catchments; multiple HUC12 catchments monitored by either a single hydrological station at the outlet or a pair of upstream and downstream stations form 6 different HUC groups that are marked as black-outlined polygons.

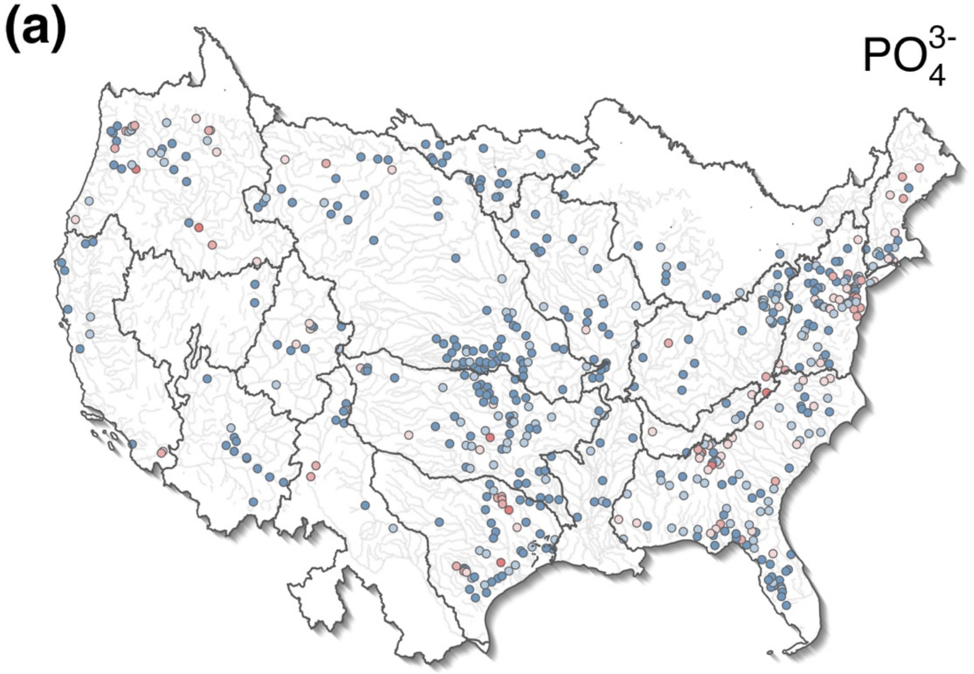

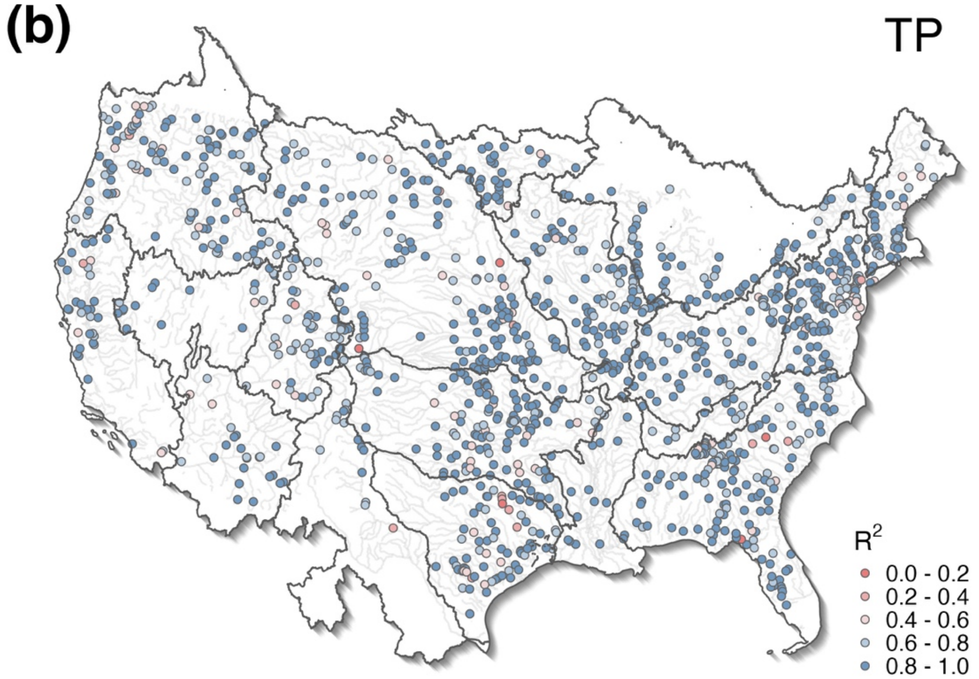

Figure S2. Coefficient of determination (r^2) of LOADEST model fits at each monitoring station for (a) PO_4^{3-} and (b) TP.

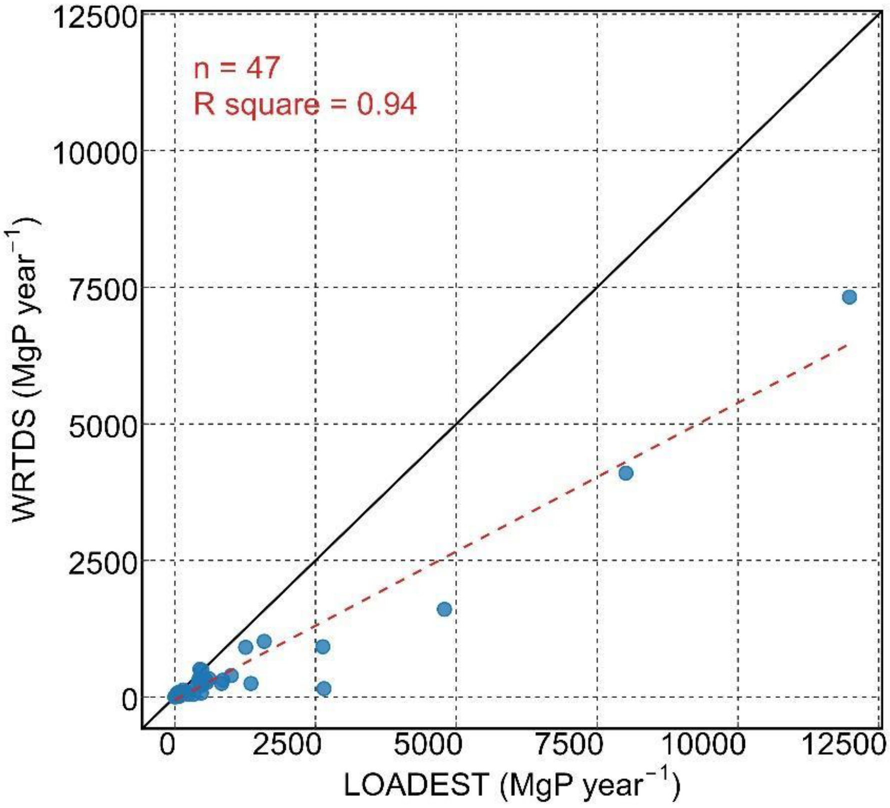

Figure S3: Comparison of riverine PO_4^{3-} loads between our estimates and previous WRTDS estimated values at 47 matched hydrological stations.

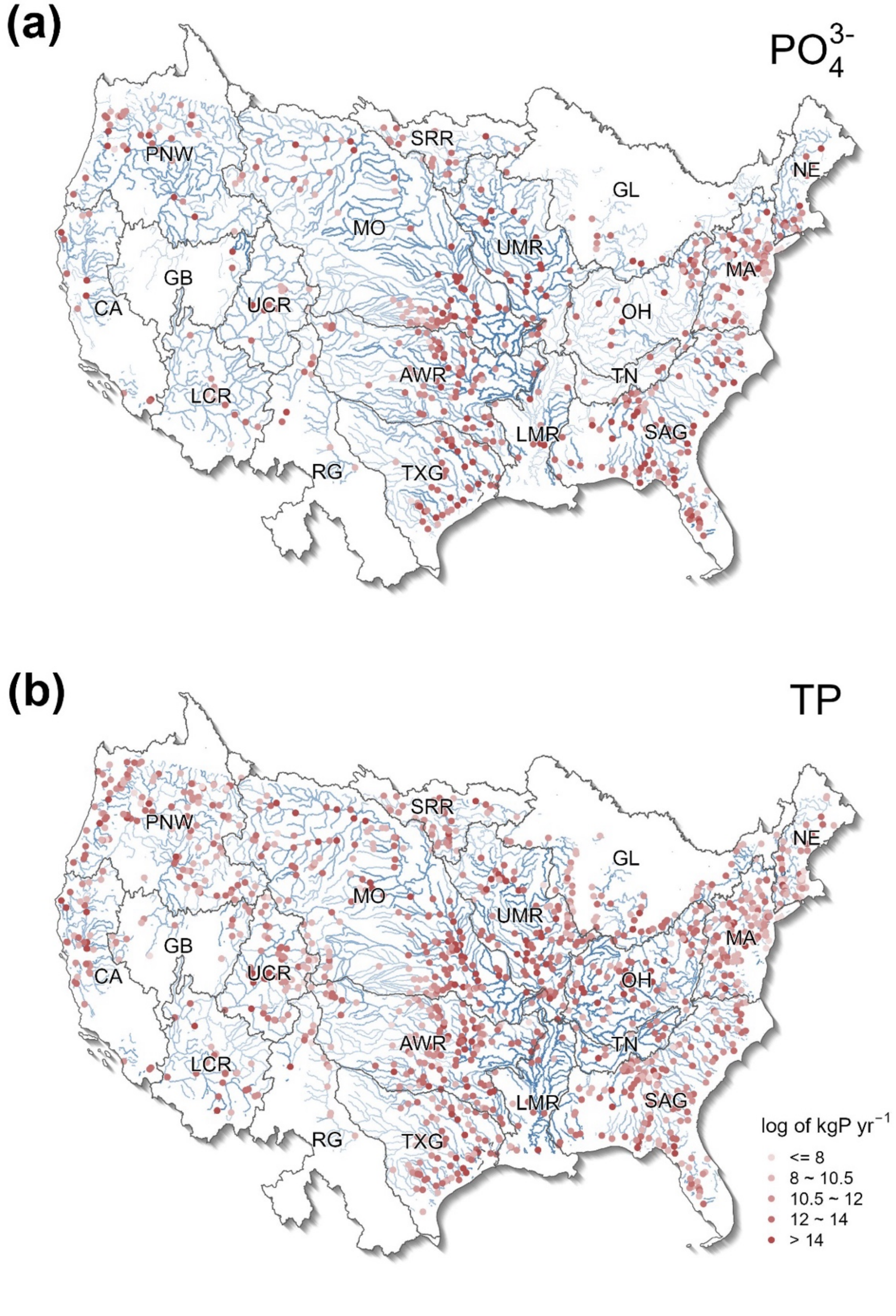

Figure S4: Riverine (a) PO_4^{3-} and (b) TP loads at 547 and 1,225 stations, respectively, across the CONUS. The boundary lines show the Hydrologic Unit Catalogue 2-digit (HUC2) watersheds. For visualization purposes, the logarithm was used here.

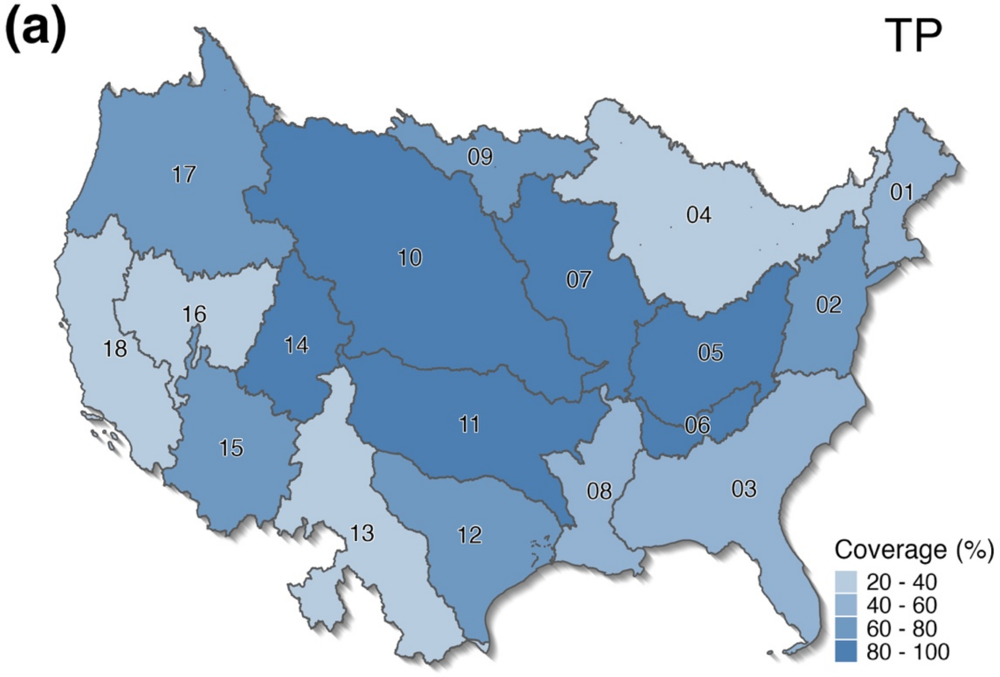

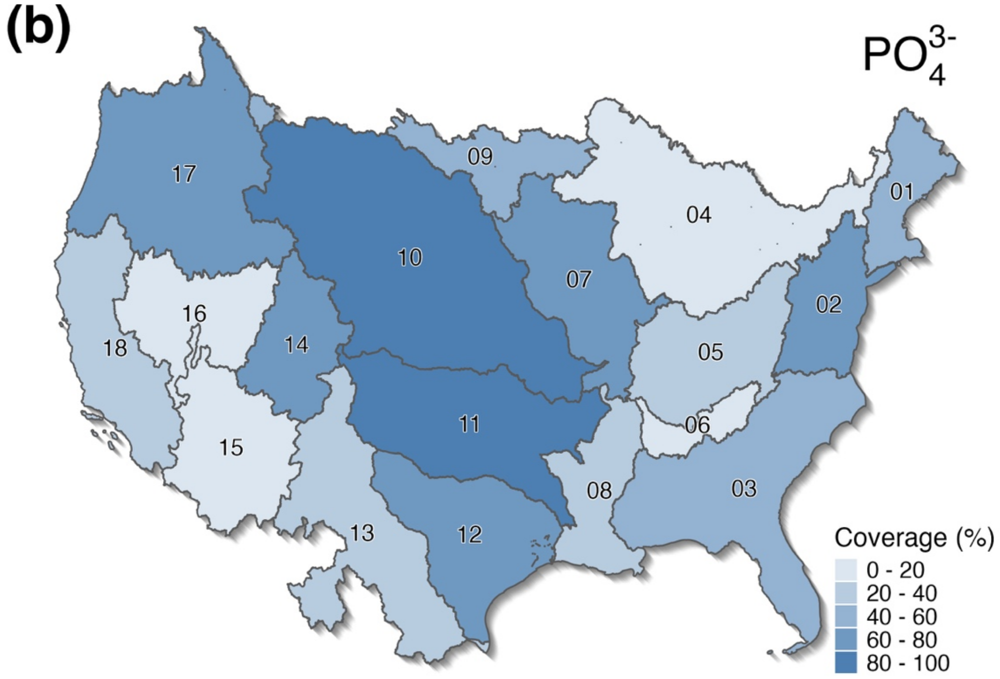

Figure S5: Spatial coverage of riverine gain and loss estimates across HUC2 basins in the CONUS for (a) TP and (b) PO_4^{3-}.

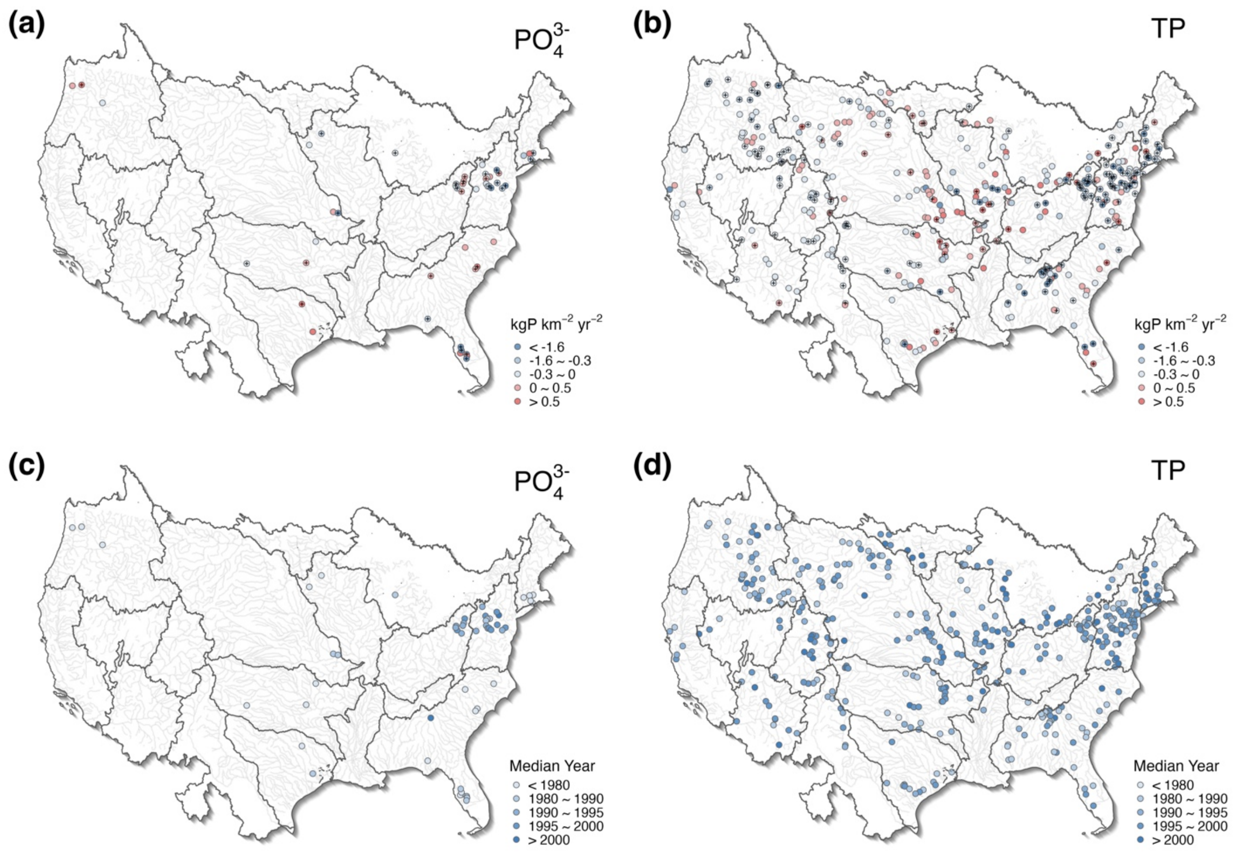

Figure S6: Spatial pattern of areal-normalized Sen's slope of riverine (a) PO_4^{3-} and (b) TP loads, and the median year of temporal coverage for (c) PO_4^{3-} and (d) TP at the monitoring stations. Crosses indicate stations with statistically significant trends ($p < 0.05$).

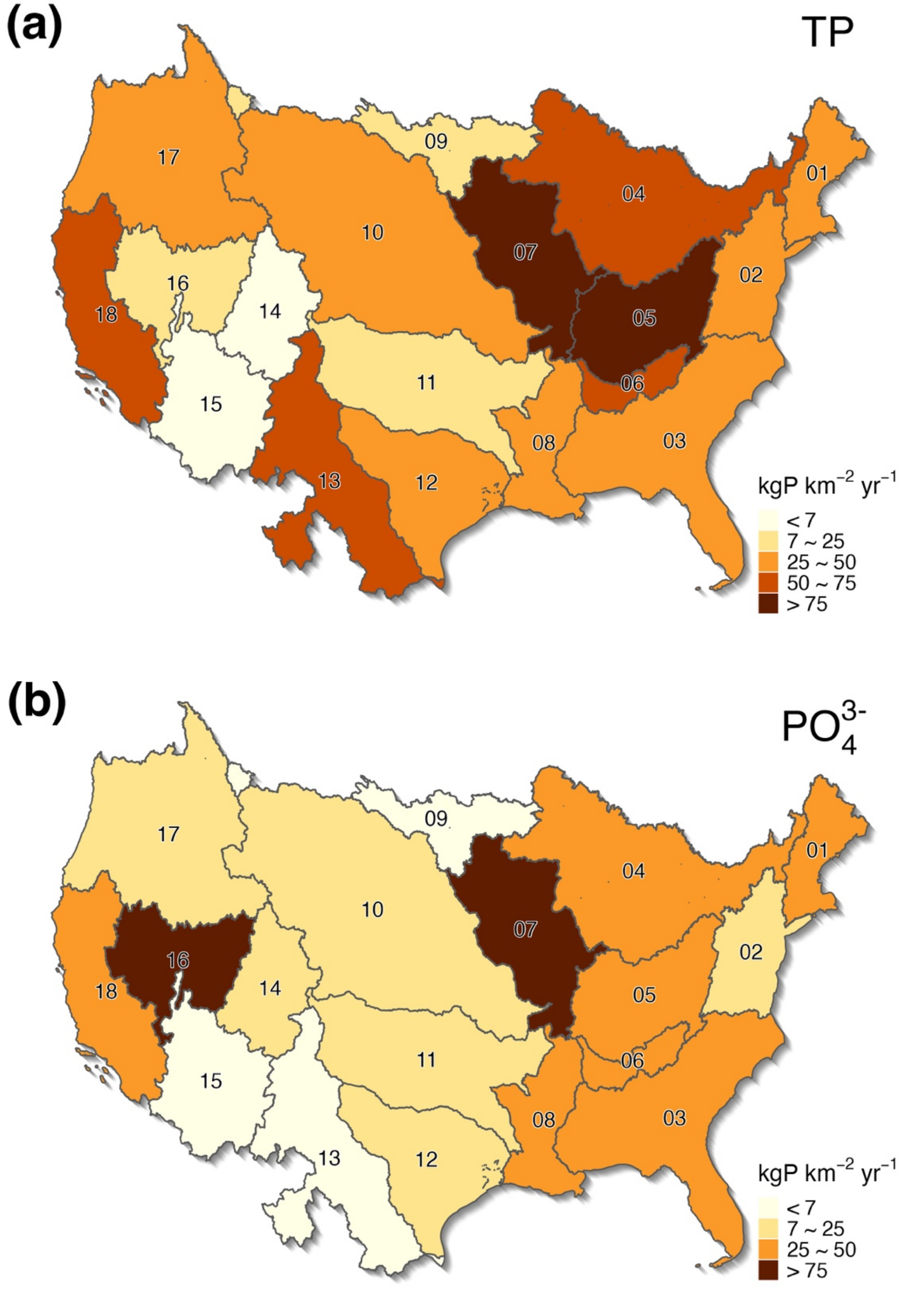

Figure S7: Area weighted average gain and loss of (a) TP and (b) PO_4^{3-} at the HUC2 level.

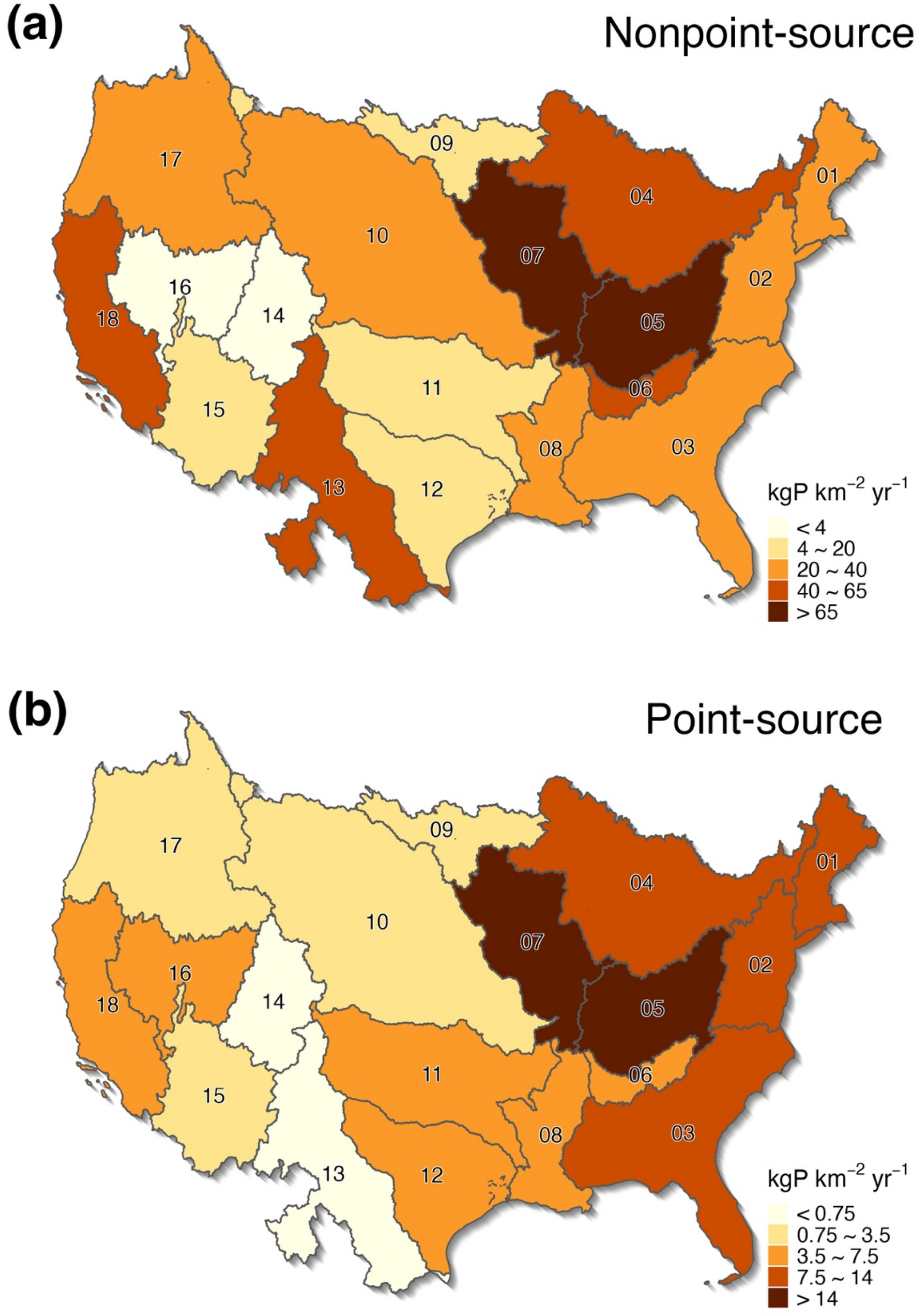

Figure S8: Area weighted average (a) non-point source and (b) point-source contributions at the HUC2 level.

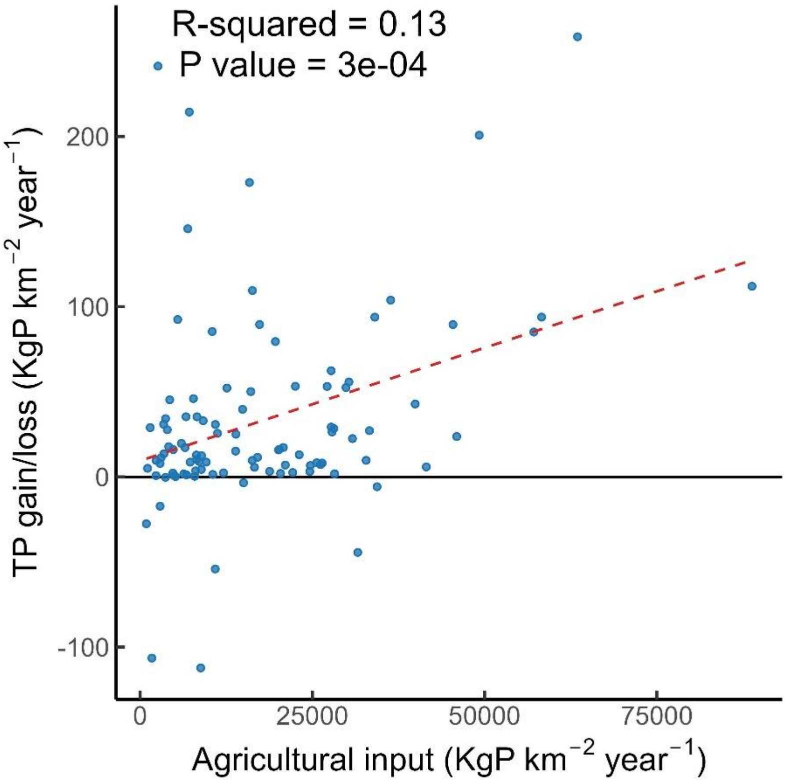

Figure S9: Scatter plot of accumulated agricultural inputs with TP gain and loss at the HUC4 level.

Table S2: Spatial coverage of riverine gain and loss estimates across HUC2 basins in the CONUS.

HUC2	TP Coverage (%)	PO_4^{3-} Coverage (%)
01	49.97	44.48
02	65.59	63.06
03	56.08	49.07
04	21.04	6.97
05	99.91	29.41
06	99.93	10.16
07	99.54	78.84
08	52.10	22.69
09	73.68	55.67
10	96.72	96.51
11	95.08	84.79
12	78.60	77.55
13	21.96	26.33
14	95.96	77.24
15	63.45	17.71
16	26.85	7.64
17	79.66	78.51
18	36.08	27.79

References

1. Runkel, R. L., Crawford, C. G., and Cohn, T. A.: Load Estimator (LOADEST): A FORTRAN program for estimating constituent loads in streams and rivers, https://doi.org/10.3133/tm4A5, 2004.
